# O4-8 Measuring capabilities for physical activity-related health outcomes: A Systematic review

**DOI:** 10.1093/eurpub/ckac094.032

**Published:** 2022-08-29

**Authors:** Maike Till, Susanne Ferschl, Karim Abu-Omar, Peter Gelius

**Affiliations:** Department of Sportscience and Sport, Friedrich-Alexander-Universität Erlangen-Nürnberg, Erlangen, Germany

**Keywords:** Capability Approach, measurement tools, systematic Review

## Abstract

**Background:**

Health promotion projects commonly measure health outcomes and behavior to provide proof of effectiveness. An alternative concept that focuses on the real opportunities a person can choose from to influence their health is Amartya Sen's capability approach. Numerous tools have been developed to measure capability change in general, but it remains unclear which ones can be applied specifically to physical activity (PA).We therefore conducted a systematic review to identify appropriate tools to measure capabilities for physical activity and health and provide information on their quality.

**Methods:**

The review included a total of 6,850 articles published between 2000 and June 2019 that were identified via searches on PubMed, EbscoHost, and ProQuest. Screenings of titles/abstracts and full texts were conducted independently by two researchers using Endnote X9 and Microsoft Excel. Identified tools are currently being analyzed regarding their indicators, evaluation methods, quality, and the extent to which they address capabilities for physical activity.

**Results:**

The screening resulted in a total of 49 articles included in the analysis. Preliminary results show a diverse use of methods for measuring capabilities for healthy lifestyles. Preliminary results show that three categories of instruments can be identified: (a) Five studies employed secondary data analysis of specific datasets to extrapolate capabilities for healthy living; (b) five articles dealt with measuring capabilities using qualitative approaches (interviews, video recordings); (c) 39 articles reported on a total of 10 different questionnaires to measure capabilities. We identified only one instrument (employing both a questionnaire and qualitative measures) that explicitly measured capabilities for PA, albeit only for a specific target group.

**Conclusions:**

The identified articles show that capabilities for healthy lifestyles are mostly measured by questionnaire. Available tools are mostly target group- and setting-specific. Currently, there is a dearth of tools that explicitly cover capabilities for PA, especially across settings or target groups. Therefore, more research is needed to work towards the development of universally applicable tools to measure PA capability.

